# Development of a Personalized Recommendation System for E-Commerce Products for Distributed Storage Systems

**DOI:** 10.1155/2022/4752981

**Published:** 2022-06-20

**Authors:** Shurui Gao, Weidong Meng

**Affiliations:** School of Economics and Management, Beijing-Tianjin-Hebei Collaborative Development Management Innovation Research Center, Yanshan University, Qinhuangdao, Hebei 066004, China

## Abstract

Because the distributed storage system is based on network technology, it can store data in multiple independent low-cost physical storage devices, and it is also suitable for large-capacity storage, so it has become more and more popular. Today, common applications of distributed storage systems include cloud storage services, data center storage services, and P2P storage services. Typical ones are GFS, HDFS, OceanStore, and Dynamo. Due to regional and economic differences, the development level of global e-commerce (b2c) is very inconsistent. b2c contains the following key tags: buying and selling, which is the core of the website platform. E-commerce provides business users with transparent information and high-quality cheap products. Logistics is the basic guarantee for customers to execute transactions, and it is also a strict indicator of the website platform. There will be many visits during the operation of the e-commerce system, and the number of users in the early stage will increase exponentially. A safe and efficient e-commerce system can provide users with one-stop transaction support and convenient transaction processes. The personalized recommendation system has formulated some rules for certain fields, based on these rules, and defined certain types of knowledge for certain items to meet the needs of certain users and use the defined reasoning rules to generate recommendation results.

## 1. Introduction

Most distributed storage system solutions are based on network storage and usually use dedicated servers and disk arrays, which makes them reliable. However, in the face of large data storage and large-scale access, it will become a difficulty for system performance and reliability. The disadvantage of the distributed storage system solution is that it is too expensive [1]. As a result, traditional Chinese network storage cannot meet the needs of today's large-scale storage applications. In recent years, with the development of international e-commerce trading platforms, people have found that the development of website has always followed its characteristics, which is geographical differences, and has gradually been imitated by companies in the market. The development of e-commerce in industrialized countries was relatively early, far ahead of many developing countries. With the development of industrialized countries, the future trend of China's e-commerce trade will gradually approach developed countries [2, 3]. For e-commerce product subsystem topologies, system storage servers and database server clusters are generally in the unit's computer room to achieve load balancing of Nginx technology, and distributors, members, and visitors can all become users. Devices that can access the Internet belong to this category [4]. The user's operation is to first connect to the main business line through the network and then reach some nodes on the connection server where it is located, and finally the Nginx server performs distribution, registration and login, product release management, product transaction and product recommendation selection, and product purchase and searches these elements of order from the subsystem of e-commerce products. The popularity and rapid development of the Internet have brought a lot of information to the public [5, 6]. With the help of e-commerce, especially in social and mobile communications, people have entered the era of data informatization. The explosive growth of information makes it difficult for people to find the information they need. Currently, the main solution to information overload is personalized recommendation system technology. Information retrieval technologies such as search engines have been very successful, but users must enter precise keywords to obtain search results. However, in some cases users often cannot accurately describe their information needs; personalized recommendation systems can be effective to solve this problem, and personalized recommendation is a hot field of current research.

## 2. Related Work

The literature introduces that the main idea of the content algorithm is to analyze the attributes and information of the tourists, find keywords and tags related to the user's taste, use this information to reconstruct the user's portrait and types of objects, and make personalized recommendations on this basis. Knowledge-based recommendation technology makes recommendations based on users and products, and derives various products that can meet their needs [7, 8]. Compared with the conventional recommendation algorithm, the recommendation algorithm does not depend on the user's historical characteristics, so there are no problems such as cold start. The literature introduces that the e-commerce platform subsystem uses JSP technology to display on the front-end page of the system, and all transactions are implemented in the trading platform functions to ensure an excellent user experience on the Internet [9]. The literature introduces that the business logic of each functional module of the product subsystem of the e-commerce platform is developed in JAVA language and uses the spring MVC development framework. The development components of the factory model and the bottom-level package of the framework are designed to meet the requirements of the actual development environment and business customization. The literature introduces e-commerce platform product subsystems that can provide secure, one-stop transaction services for enterprise users. This is after an in-depth investigation of the current mainstream e-commerce platform, and then a standardized software development process with high security and excellent experience has been researched [10, 11]. The literature introduces that the product subsystem of the e-commerce platform has been successfully applied to production, and the daily transaction volume of the product subsystem is about 1 million yuan. It is necessary to develop the product subsystem of the mobile phone version, but the security needs to be continuously optimized and updated [12–14].

## 3. Model Design for Distributed Storage Systems

### 3.1. Distributed Storage System

#### 3.1.1. System Features

*(1) Scalability*. A fully distributed storage system cluster can contain thousands of nodes. This number may be very large. During use, the resources of the cluster will become smaller and smaller as the amount of data increases. If the resources are exhausted, it will no longer be able to provide services to the cluster. The distributed storage system is highly scalable. The system can be expanded horizontally by simply adding nodes, and the overall system performance will be further improved.

*(2) Fault Tolerance*. Distributed storage systems have the property of solving failures and can realize automatic fault tolerance through software services. If data are lost or there is a problem with the node, the data can be restarted after restarting the node.

*(3) High Performance*. Distributed storage systems can provide fast response and feedback services to other applications, and the system performance is good. Generally, upper-layer applications built on distributed storage systems will show high performance. Integrating hundreds of nodes together to form a large cluster, the performance is naturally guaranteed.

*(4) Ease of Use*. As a basic component, the distributed storage system must be connected to other top-level applications to provide corresponding services. Because there are many different types of top-level applications, the distributed storage system must provide different interfaces for applications on each platform, which developers can learn in time.

#### 3.1.2. Key Technologies

*(1) Metadata Technology*. In a distributed storage system, I/O operations are the most commonly used operations and are also very resource-consuming operations. Distributed means decentralized data storage and decentralized I/O operations. When saving a file, use the file as a unit or divide the file into multiple slices, and then save them in the slices. These two storage methods are widely used. In the former, files are stored on different nodes, while in the latter, multiple pieces of files are stored on different nodes. Therefore, no matter which way, the positioning of the data will become the primary issue.

The metadata server or master node is established using the mapping relationship between logical and physical addresses, and the metadata information can be used to find files more quickly. Sending a request to the metadata node is the beginning of the I/O operation. The metadata node response is all types of information in the cluster, including information such as size and location. Using location information, you can quickly find files on the cluster and perform appropriate I/O operations after finding the files, which helps improve system performance.

*(2) Copy Technology*. Safety and reliability are always the first. For example, all kinds of errors, software problems, and hardware problems will threaten the security and reliability of data, among which there are many unforeseen problems. When these problems occur, data are usually lost. At this time, the user cannot access data or other operations. In addition, data usually have very important value. The loss caused by data loss is huge. Most traditional data storage is single-copy storage. When something goes wrong, the data will disappear forever, but more advanced systems use copy technology to indirectly back up files.

Distributed data storage systems use replication technology to store data in copies to improve security. Data redundancy is valuable. Copy technology is scattered and extensive. The best way is to make copies of files, keep the duplicate copies in a decentralized state, and then distribute them to individual data nodes on the system. It will not affect each other and guarantee the security and reliability of the data. If a node fails, data can also be accessed from other nodes. After restarting the failed node, the system can detect the problematic data through an automatic recovery mechanism and copy data from other nodes with the same data, ensuring that all copies in the system are in the correct state.

#### 3.1.3. Data Repair Model

The optimization goal of the heterogeneous repair problem is to minimize the total repair cost C, that is, the total cost required to download data from the remaining storage nodes in a strip. Design the following heterogeneous repair optimization model for RDP code:

(1) $$\begin{array}{c} \text{Minimize }C=\sum_{i=0,i\neq t}^{p}c_{i}y_{i}. \end{array}$$

The constraint condition of the above optimization model is that the data blocks lost on the faulty nod*e V* in each stripe can be recovered correctly. For RDP encoding, a feasible repair plan needs to meet:

(2) $$\begin{array}{c} \sum_{i\neq t}y_{i}\geq \frac{3{\left(p-1\right)}^{2}}{4},\\ \frac{p-1}{2}\leq y_{i}\leq p-1i\neq t. \end{array}$$

In fact, a repair sequence provides information such as the download distribution {*y*_*o*_,…, *y*_*t*−1_, *y*_*t*+1_,…, *y*_*p*_} and the specific data check block will be downloaded.

(3) $$\begin{array}{c} y_{j}=\left(p-1\right)-\left(\sum_{i=0}^{p-1}x_{i}-\sum_{i=0}^{p-1}x_{i}x_{\left(i+j-t{>}_{p}\right)}\right). \end{array}$$

Consider the repair sequence that minimizes the amount of data download; that is, exactly (*p* − 1)/2 faulty blocks need to be repaired by using a diagonal check set.

(4) $$\begin{array}{c} y_{j}=\frac{\left(p-1\right)}{2}+\sum_{i=0}^{p-1}x_{i}x_{<i+j-k>p}. \end{array}$$

Introducing the data download distribution represented by equation (4) into the heterogeneous repair optimization model of equation (1), then the total repair cost *C* required for the repair process can be expressed as follows:

(5) $$\begin{array}{c} \sum_{j\neq t}w_{j}y_{j}=\sum_{j\neq t}\left(\frac{\left(p-1\right)}{2}+\sum_{i=0}^{p-1}x_{i}x_{\left(+j-r{>}_{p}\right)}\right)\\ w_{j}=\frac{p-1}{2}\sum_{j\neq 1}w_{j}+\sum_{j\neq 1}\sum_{i=0}^{p-1}x_{i}x_{\left(i+j-t>p\right)}w_{j}. \end{array}$$

The first half of equation (5) is a fixed value. Therefore, it is only necessary to consider minimizing the second part. Therefore, the heterogeneous repair model can be reformulated as follows:

(6) $$\begin{array}{c} \text{Minimize}\sum_{j\neq k}\sum_{i=0}^{p-1}x_{i}x_{\left(i+j-k{>}_{p}\right)}w_{j}\\ \text{subjectto}\sum_{i=0}^{p-1}x_{i}=\frac{\left(p-1\right)}{2}x_{i}\in \left\{0,1\right\}\text{for}i=0,1,\ldots ,p-2x_{p-1}=0. \end{array}$$

Because the missing data block of the last line of the repair sequence, that is, the imaginary line, must use the line check set to repair the faulty data, *x*_*p*1_′ = 0 is required. Therefore, the repair sequence with *x*_*p*1_′ = 0 is not feasible.

(7) $$\begin{array}{c} y_{j}^{\prime }=\frac{\left(p-1\right)}{2}+\sum_{i=0}^{p-1}x_{i}^{\prime }x_{\left(+j-k{>}_{p}\right)}^{\prime }\\ =\frac{\left(p-1\right)}{2}+\sum_{i=0}^{p-1}x_{\left(i+r>p\right)}x_{\ll <i+r>p+j-k{>}_{p}}. \end{array}$$

Now, calculate the repair cost *C* of *R* = {1110000}. It can be verified that the agent node will download 3 data blocks from storage nodes 3, 4, and 7, respectively; download 4 data blocks from storage nodes 2 and 5; and download 5 data blocks from storage nodes 1 and 6 to complete the repair task. Therefore, the total repair cost *C* is

(8) $$\begin{array}{c} \frac{5\alpha }{68}+\frac{4\alpha }{109}+\frac{3\alpha }{110}+\frac{3\alpha }{86}+\frac{4\alpha }{110}+\frac{5\alpha }{10}+\frac{3\alpha }{113}=0.7353\alpha \left(\text{in sec}\right). \end{array}$$

Minimize the amount of data download repair sequence and repeat the above steps. Finally, the minimum cost repair sequence found by CHR is *R*∗ = {1010100}. Therefore, the final minimum repair cost *C*∗ is

(9) $$\begin{array}{c} \frac{3\alpha }{68}+\frac{5\alpha }{109}+\frac{4\alpha }{110}+\frac{4\alpha }{86}+\frac{5\alpha }{110}+\frac{3\alpha }{10}+\frac{3\alpha }{113}=0.5449\alpha . \end{array}$$

The CHR algorithm significantly reduces the repair cost brought about by traditional repair. Using the traditional repair algorithm, the agent node needs to download 6 data blocks from node 1 to node 6, respectively. The final repair cost is

(10) $$\begin{array}{c} \frac{6\alpha }{68}+\frac{6\alpha }{109}+\frac{6\alpha }{110}+\frac{6\alpha }{86}+\frac{6\alpha }{110}+\frac{6\alpha }{10}=0.9221\alpha \left(\text{in sec}\right). \end{array}$$

Then, the total repair cost of its heterogeneous repair is

(11) $$\begin{array}{c} \sum_{i=0,i\neq t}^{n}c_{i}y_{i}.\; \end{array}$$

#### 3.1.4. Algorithm Simulation Experiment

In this section, simulation experiments will be used to quantitatively evaluate the advantages of the proposed CHR algorithm compared with traditional repair and hybrid repair algorithms in terms of computational complexity and fault repair performance.

The simulation experiment considers the star network topology (as shown in Table 1).

The comparison results are given in Table 2. The second column of the table gives the probability that the repair sequence returned for different *p* value CHRs can provide the global minimum repair cost; the third column gives the maximum improvement of the global minimum repair cost for CHR. It is observed that the repair sequence returned by the CHR algorithm provides the global minimum repair cost with a high probability of over 93%. In addition, the global minimum repair cost is at most 6.46% less than the repair cost provided by the repair sequence returned by the CHR. Therefore, it can be concluded that the CHR algorithm can provide a global optimal repair solution with a high probability under the premise of significantly reducing the search time.

**Figure 1:**
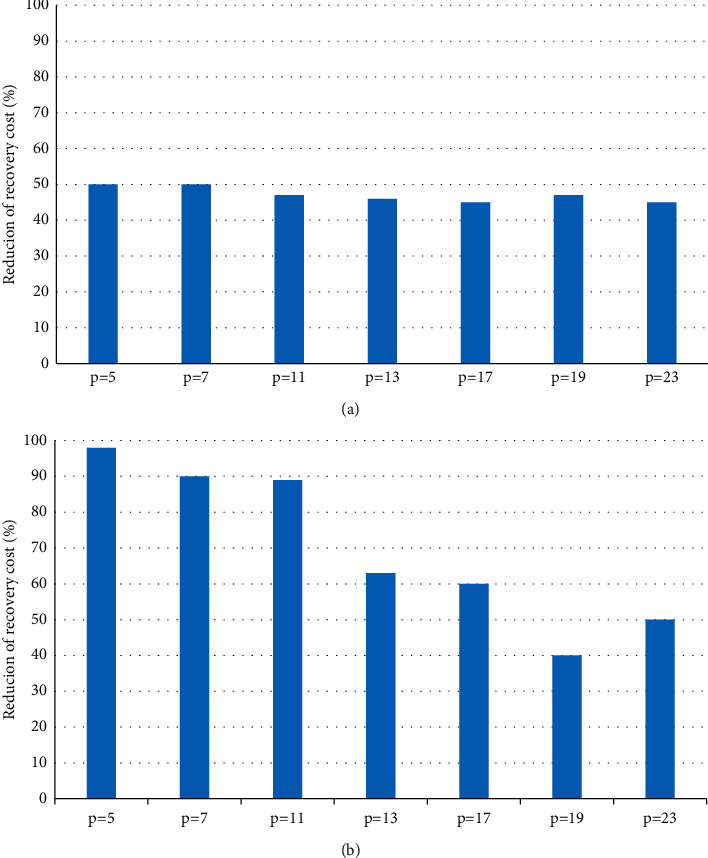
Performance comparison of CHR repair, traditional repair, and hybrid repair. (a) CHR repair vs. conventional repair. (b) CHR repair vs. conventional repair.

Figure 1 shows the improvement results of CHR repair compared to traditional repair and hybrid repair. It is observed that when *p* = 5, the CHR algorithm can reduce the repair cost of traditional repair by up to 50% and the hybrid repair algorithm by up to 30%.

**Figure 2:**
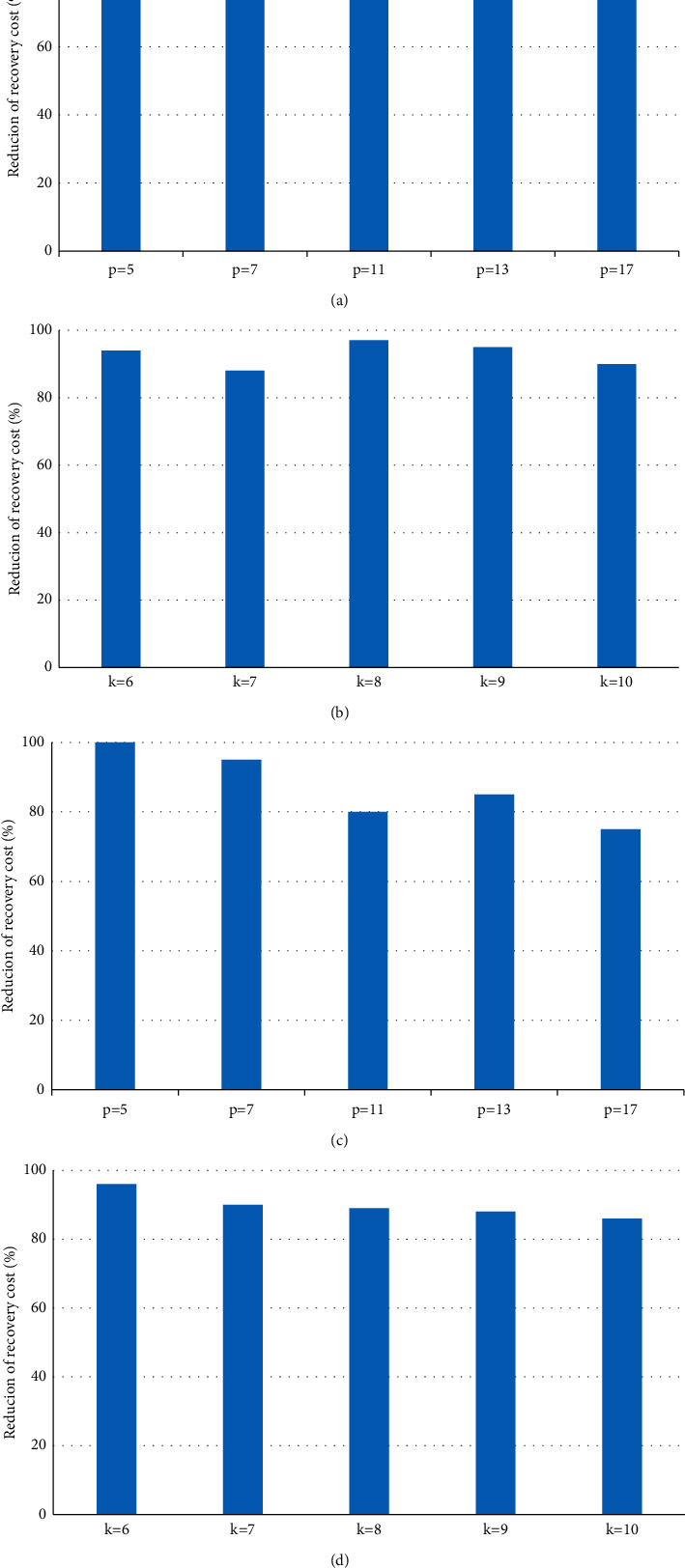
The repair cost reduction ratio of HeRR compared with conventional ((a) and (b)) and HoRR ((c) and (d)). (a) STAR, over conventional. (b) CRS (*m* = 3, *w*=4), over conventional. (c) STAR, over HoRR. (d) CRS (*m* = 3, *w*=4), over HoRR.

Figure 2 shows the reduction ratio of the single-node fault repair cost of HeRR repair compared with conventional repair and HoRR repair. As shown in Figures 2(a) and 2(b), compared with conventional repair, HeRR repair can reduce the repair cost by about 40% and 30% for STAR code and CRS code, respectively. As shown in Figures 2(c) and 2(d), compared with HoRR repair, HeRR repair reduces the repair cost by about 20% and 10% for STAR code and CRS code, respectively.

### 3.2. E-Commerce Product Personalized Recommendation Technology

#### 3.2.1. Recommendation Model

The root of content recommendation is filtering information. It is possible to record the content of things that tourists are interested in for recommendation. The commonly used algorithm is TF-IDF. There are *N* documents, of which *ni* documents contain the keyword *k*_*i*_, *f*_*ij*_ is the number of times the keyword *k*_*i*_ appears in the text *dj*, and the word frequency *ki* in the document *d*_*j*_ is defined as *TF*_*ij*_.

(12) $$\begin{array}{c} TF_{ij}=\frac{f_{ij}}{\underset{z}{\text{max}}f_{zj}}. \end{array}$$

The denominator represents the frequency of the most common keyword *k*_*z*_ in the document *d*_*j*_. The more the frequency and the lower the frequency in other documents, the more important the keyword becomes. The inverse document frequency of the keyword *k*_*i*_ is defined as

(13) $$\begin{array}{c} I\;DF_{i}=\text{log}\frac{N}{n_{i}}. \end{array}$$

The importance of *k*_*i*_ to the text *d*_*j*_*w*_*ij*_ is defined as

(14) $$\begin{array}{c} W_{ij}=TF_{ij}*I\;DF_{i}. \end{array}$$

**c** ^⟶^ represents the user's model, represents the content description of the document, and usually calculates the angle of the cosine vector as a utility function:

(15) $$\begin{array}{c} u\left(c,d\right)=\text{cos}\left(\overset{⟶}{W_{c}},\overset{⟶}{W_{d}}\right)\\ =\frac{\sum_{i=1}^{K}W_{ci}W_{i\;d}}{\sqrt{\sum_{i=1}^{K}W_{ci}^{2}}\times \sqrt{\sum_{i=1}^{K}w_{i\;d}^{2}}}. \end{array}$$

In addition to the methods of obtaining information, content-based recommendations can also use many machine learning techniques. The probability that the user browses the web page *I* belongs to the category *C*_*i*_:

(16) $$\begin{array}{c} P\left(C_{i}|I\right)=\frac{P\left(C_{i}\right)}{P\left(I\right)}\prod_{j}P\left(k_{j}|C_{i}\right). \end{array}$$

Pearson correlation coefficient:

(17) $$\begin{array}{c} \text{Sim}\left(u,v\right)=\frac{\sum_{i\in I_{u\cap^{}I_{v}}}\left(r_{u,i}-{\bar{r}}_{u}\right)\cdot \left(r_{v,i}-{\bar{r}}_{v}\right)}{\sqrt{\sum_{i\in I_{u}\cap^{}I_{v}}{\left(r_{u,i}-{\bar{r}}_{u}\right)}^{2}}\cdot \sqrt{\sum_{i\in I_{u}\cap^{}I_{v}}{\left(r_{v,i}-{\bar{r}}_{v}\right)}^{2}}}. \end{array}$$

Cosine similarity:

(18) $$\begin{array}{c} \text{Sim}\left(u,v\right)=\text{cos}\left(\overset{⟶}{u},\overset{⟶}{v}\right)\\ =\frac{\overset{⟶}{u}\cdot \overset{⟶}{v}}{\left\|\overset{⟶}{u}\right\|\cdot \left\|\overset{⟶}{v}\right\|}=\frac{\sum_{i\in I_{u}\cap^{}I_{v}}}{\sqrt{\sum_{i\in I_{u}\cap^{}I_{v}}r_{u,i}^{2}\cdot \sum_{i\in I_{u}\cap^{}I_{v}}r_{v,i}^{2}}}. \end{array}$$

Corrected cosine similarity:

(19) $$\begin{array}{c} \text{Sim}\left(u,v\right)=\frac{\sum_{i\in I_{u\cap^{}I_{v}}}\left(r_{u,i}-{\bar{r}}_{i}\right)\cdot \left(r_{v,i}-{\bar{r}}_{i}\right)}{\sqrt{\sum_{i\in I_{u}\cap^{}I_{v}}{\left(r_{u,i}-{\bar{r}}_{i}\right)}^{2}}\cdot \sqrt{\sum_{i\in I_{u}\cap^{}I_{v}}{\left(r_{v,i}-{\bar{r}}_{i}\right)}^{2}}}. \end{array}$$

Aggregating user *C*'s *K* most similar user reviews for **Item** · **s** can predict user *C*'s score for $\mathrm{Item}\cdot s\hat{r}c$:

(20) $$\begin{array}{c} {\hat{r}}_{c,s}=a\underset{c^{\prime }\in U}{\text{gg}r}r_{c^{\prime },s}. \end{array}$$

Commonly used aggregation functions for user-based collaborative filtering recommendation algorithms are as follows:

(21) $$\begin{array}{c} {\hat{r}}_{\text{c},\text{s}}=\frac{1}{k}\sum_{c^{\prime }\in U}r_{c^{\prime },s},\\ {\hat{r}}_{\text{c},\text{s}}=\frac{1}{\sum_{c^{\prime }\in U}\left|\text{sim}\left(c,c^{\prime }\right)\right|}\sum_{c^{\text{'}}\in U}\text{sim}\left(c,c^{\prime }\right)\cdot r_{c^{\prime },s},\\ {\hat{r}}_{\text{c},\text{s}}={\bar{r}}_{c}+\frac{1}{\sum_{c^{\prime }\in U}\left|\text{sim}\left(c,c^{\prime }\right)\right|}\sum_{c^{\text{'}}\in U}\text{sim}\left(c,c^{\prime }\right)\cdot \left(r_{c^{\text{'}},s}-{\bar{r}}_{c^{\prime }}\right). \end{array}$$

Pearson correlation coefficient:

(22) $$\begin{array}{c} \text{Sim}\left(i,j\right)=\frac{\sum_{u\in U_{i}\cap^{}U_{j}}\left(r_{u,i}-{\bar{r}}_{i}\right)\cdot \left(r_{u,j}-{\bar{r}}_{j}\right)}{\sqrt{\sum_{u\in U_{i}\cap^{}U_{j}}{\left(r_{u,i}-{\bar{r}}_{i}\right)}^{2}}\cdot \sqrt{\sum_{u\in U_{i}\cap^{}U_{j}}{\left(r_{u,j}-{\bar{r}}_{j}\right)}^{2}}}. \end{array}$$

Cosine similarity:

(23) $$\begin{array}{c} \text{Sim}\left(i,j\right)=\text{cos}\left(\overset{⟶}{ı},\overset{⟶}{j}\right)\\ =\frac{\overset{⟶}{i}\overset{⟶}{j}}{\left\|\overset{⟶}{i}\right\|\cdot \left\|\overset{⟶}{j}\right\|}=\frac{\sum_{u\in U_{i}\cap^{}U_{j}}r_{u,i}\cdot r_{u,j}}{\sqrt{\sum_{u\in U_{i}\cap^{}U_{j}}r_{u,i}^{2}}\cdot \sqrt{\sum_{u\in U_{i}\cap^{}U_{j}}r_{u,j}^{2}}}. \end{array}$$

Corrected cosine similarity:

(24) $$\begin{array}{c} \text{Sim}\left(i,j\right)=\frac{\sum_{u\in U_{i}\cap^{}U_{j}}\left(r_{u,i}-{\bar{r}}_{u}\right)\cdot \left(r_{u,j}-{\bar{r}}_{u}\right)}{\sqrt{\sum_{u\in U_{i}\cap^{}U_{j}}{\left(r_{u,i}-{\bar{r}}_{u}\right)}^{2}}\cdot \sqrt{\sum_{u\in U_{i}\cap^{}U_{j}}{\left(r_{u,j}-{\bar{r}}_{u}\right)}^{2}}}. \end{array}$$

By aggregating the ratings of the *K* items most similar to **Item** · **s**, we can predict user c's ratings of **Item**$s\hat{r}\cdot c$:

(25) $$\begin{array}{c} {\hat{r}}_{c,s}=\underset{s^{\text{'}}\in I}{a\text{gg}r}r_{\text{c},s^{\prime }}. \end{array}$$

*I* is a collection of *K* items that have been evaluated by user *c* and most similar to **Item** · **s**. *rrc*, *s*′ represents user c's rating of the item *s*′. The commonly used aggregation functions are as follows:

(26) $$\begin{array}{c} {\hat{r}}_{\text{c},\text{s}}=\frac{1}{k}\underset{s^{\prime }\in I}{\sum }r_{\text{c},s^{\prime }},\\ {\hat{r}}_{\text{c},\text{s}}=\frac{1}{\sum_{s^{\prime }\in I}\left|\text{sim}\left(s,s^{\prime }\right)\right|}\underset{s^{\text{'}}\in I}{\sum }\text{sim}\left(s,s^{\prime }\right)\cdot r_{c,s^{\prime }}. \end{array}$$

#### 3.2.2. Algorithm Accuracy Analysis

**Figure 3:**
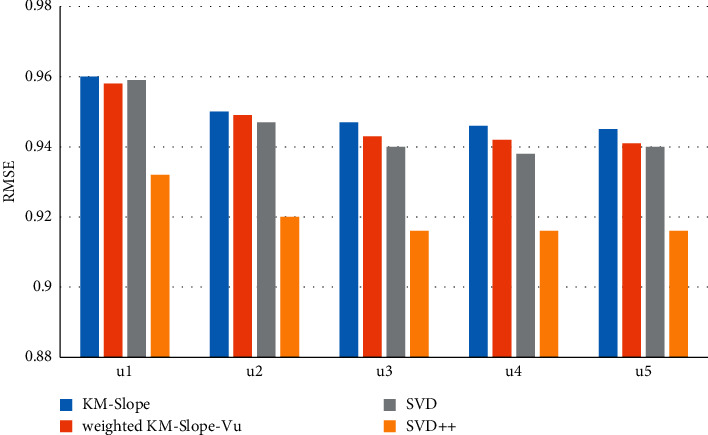
Comparison of recommended algorithm accuracy (MovieLens-100k).

Figure 3 shows the comparison of prediction accuracy performance of KM-Slope-Vu, Weighted-KM-Slope-Vu, and SVD. It is found that the average root mean square error (RMSE) of the five experiments of the KM-Slope-Vu algorithm on the MovieLens-100k data set is 0.96284, and the average root mean square error (RMSE) of the five experiments on the MovieLens-1M data set is 0.9233.

**Figure 4:**
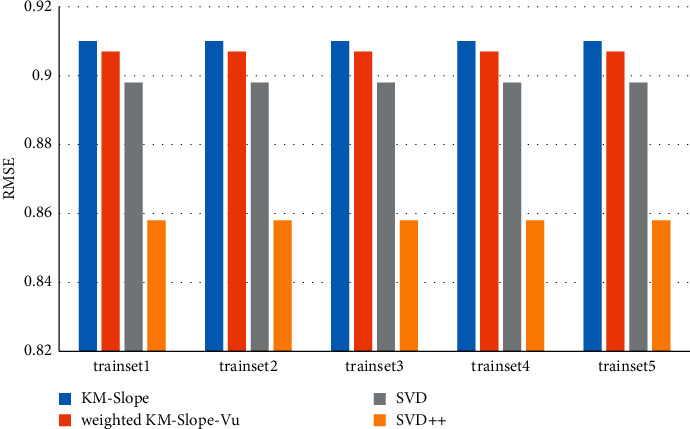
Comparison of recommended algorithm accuracy (MovieLens-1M).

The average root mean square error (RMSE) of the five experiments of the Weighted-KM-Slope-Vu algorithm on the MovieLens-100k data set is 0.95787, and the RMSE of the five experiments on the MovieLens-1M data set is 0.9192, obviously, Weighted-KM-Slope-V algorithm. The accuracy of KM-Slope-Vu is better than that of KM-Slope-Vu, because Weighted-KM-Slope-Vu takes into account the number of times the user evaluates each item. The longer the evaluation time, the more reliable it is, the more users like it, and the greater the contribution to the rating deviation and rating, which makes the poor ratings of *Itemi* and devi, *j*, which leads to more accurate predictions, as shown in Figure 4.

Analyze Weighted-KM-Slope-Vu, SVD, and SVD++ algorithms; see Table 3.

The meanings of the symbols in Table 3 are explained as follows:

 
*k*: The number of K-means output clusters;
 
*t*: The number of clustering iterations
 
*f*: The number of features used for clustering (user portrait: gender, age, occupation, etc.)
 
*m*: Number of users
 
*n*: Number of items
 
*d*: Decompose the user rating matrix *Rm* × *n* into two low-rank matrices *Pm* × *d* and *Q*  *d* × *n*

## 4. Design and Practical Application of Personalized Recommendation System for 4b2c E-Commerce Products

### 4.1. System Requirement Analysis

#### 4.1.1. Analysis of Functional Requirements of Product Subsystems

*(1) System Requirements*. A system needs to be designed that integrates members who purchase products into the transaction process, and merchants disclose product matching transactions. The system not only supports product ordering, but also enables members to postpurchase information, adjust dealer quotations, and accept member quotations.

*(2) Functional Requirements*. ① Support members and affiliated companies when registering this system. ②Support searching products and stores by keywords. ③ Click the product category to support product search. ④ Click the product sample or business logo to support the request for product and business information. ⑤ Support to browse product information such as product-specific information, product name, product price, product price tracking range change area, product display photos, product sales, and product classification information. ⑥ We support price determination based on the purchase quantity. Help support members to add products to orders and trade with distributors. ⑦ Provide assistance when members add products to orders and trade with sellers. ⑧ Display order status information to members. ⑨ It is convenient for sellers to check the status of order information and give the process introduction in time. ⑩ Provide assistance when the buyer and seller check the logistics.

#### 4.1.2. Analysis of Nonfunctional Requirements of Product Subsystems

1. Technical requirements: choose Spring MVCt back-end development framework and front-end development combination of html + css + JavaScript + jquery, and search module chooses lucence search engine.
2. Performance requirements: This system must support the registration of at least 20 W members. According to the company's business estimates, the number of user visits in the daily product statistics is about 100,000. According to a survey of the society and the company, the load timeout of the entire process from the user request to the page load is 3000 milliseconds. After that, 82% of the members felt that the reaction speed was relatively slow. Judging from the situation, the homepage, detailed information, and search pages are more concerned by member users. The maximum time can reach 999 milliseconds, and the page is 2999 milliseconds. When there is only one server, the processing request capacity of 1001TPS is the lowest.
3. Reliability requirements: The system must be very reliable. If the system fails prematurely, Tomcat must be restarted in time. If Tomcat cannot be restarted, the system operation and maintenance personnel should be notified to intervene immediately by e-mail and SMS, and the incorrect information should be registered for later correction.
4. Practical requirements: When the system functions are complete, it is necessary to do a good job monitoring. The ultimate goal of the entire system operation is to achieve convenience, simplicity, and efficiency. Members can not only easily retrieve, view, add, and place orders, but also simplify business product management and order processing.
5. Security requirements: In the product subsystem of the e-commerce platform, security is always the first. Whether he is a member or an administrator, he should provide services, including transmission. Therefore, system development uses various key technologies to prevent intrusion and information theft, such as B database encryption, network gates, system firewalls, CA certificates, and communication signal encryption.

### 4.2. Travel E-Commerce Recommendation System Design

The entire system architecture of the e-commerce platform includes front-end product subsystems that form the core of the document, as well as member management, system management, suggestion, and complaint systems. All data within the scope of the platform will also be provided. For example, statistical analysis, back-end ERP, CRM, and other management systems all have large-scale framework systems.

**Figure 5:**
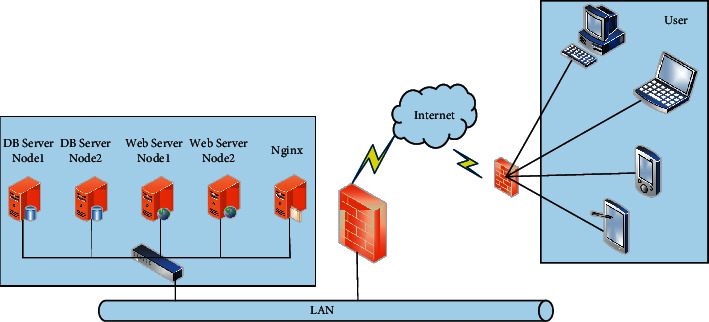
Topological structure diagram of product subsystem.

For the product subsystem of topology, the server and database are basically set in the computer room of the unit, and Nginx technology is generally used to achieve load balancing. Users are divided into dealers, members, and visitors. All devices with Internet access rights and user requirements can be referred to as access categories. They operate on the device through the network and then are connected to the main line. After connecting to some nodes on the server, they are finally distributed by the Nginx server.  Figure 5 shows the network topology.

**Figure 6:**
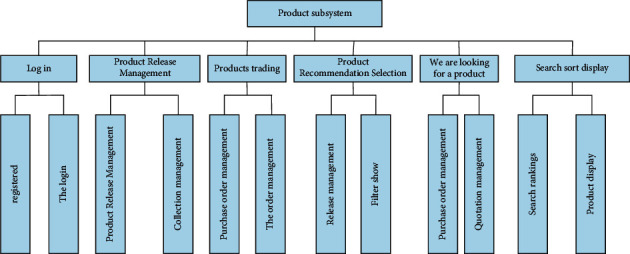
Functional structure diagram of product subsystem.

Registration and login, product release management, product transaction, product recommendation selection, product purchase, and search order display, these six departments jointly build the product subsystem.  Figure 6 is very intuitive to reflect this function and specific structure. This picture clearly shows some of its basic functions and main functions, so that everyone can see at a glance.

### 4.3. Database Design

Especially in the enemy behind the design of this subsystem, it is necessary to fully consider whether the rationality of the field types and entity classes meet the requirements. The table structure of the product is shown in Table 4.

When the database was designed, some random reasons were fully thought of, and adjustments were made immediately. This is the detailed design of the main database table, as shown in Table 5.

The core business data of the B2B system are order and product. Buyers first choose products when they purchase goods and then become orders, and finally complete the transaction, as shown in Table 6.

Merchants deliver goods in a targeted manner based on the demand information sent by buyers. After the final transaction is successful, the transaction amount is received. All the goods are provided by the merchant, and then they are delivered to logistics for transportation. Buyers get the final product from logistics, as shown in Table 7.

### 4.4. System Test

#### 4.4.1. Test Environment

The company's test environment is a dedicated test machine in the company's server room. In JS method verification, JS code specification, online monitoring, URL testing, and front-end code performance analysis, JSHint must be used as a web testing tool. The main function of JSHint is to check the front-end code. The first step is to download the Eclipse JSHint plug-in, which is also a prerequisite, then write the required script projects in, and finally start the test.

There are already many mature open-source libraries available for website performance testing. The system uses LoadRunner to automatically check the website. The test plan includes the number of resources on each page, loading time, especially the user experience is a very important indicator, and finally the integration gives a reasonable opinion.

#### 4.4.2. Test Results

The product subsystem is a website system. Everyone knows that on the same JSP or HTML page, different browser versions, types, kernels, and other issues may produce different results. Methods such as bootstrap are not compatible, and the browser is not fully adapted to exist in some JS libraries (such as jQuery). Therefore, compatibility testing should verify all types of kernel browsers and provide inapplicable solutions.

After fully running the above test and checking all front-end pages of the e-commerce platform product subsystem in each browser, the subsystem products meet the requirements for successful display on IE, Chrome, Safari, Opera, Firefox, and other platforms. At the same time, JS code can also be run in a standardized manner.

1. The physical environment for performance testing. The test planning system and the database server have the same physical conditions and are also located in the company's computer room. The network environment is consistent with other servers, so that the network status of the server can be best simulated under actual conditions. Only when the network environment and infrastructure are perfect, can the maximum throughput and the maximum negative endurance of the test system be simulated.
2. The overall plan for stress testing. The server being tested for performance this time is the system server of the Tomcat deployment project. Under normal circumstances, the most basic test structure can be constructed after printing test, system, business, interface, and local database server. The stress test method includes the stress test server by continuously opening, closing, clicking, and jumping to the page on the system server to test the stress tolerance of the project hosted on the system server.
3. I have used the Siege framework before stress testing the application. It is mainly a method of self-checking by following the script. It is also possible to combine this test tool with the actual environment of the unit. Testers also understand the test points and general test levels, and can use the test framework appropriately. After commissioning and training employees, the number has further reached 9,999/s or more. After the framework integration modification and improvement, in theory, everyone has accepted the standard of the stress test server.

When checking the throughput of the system, we generally use infrastructure and networks. The main content is to increase the number of simultaneous postings. Start with the first connection requested, and then gradually increase. At this time, you can find that the corresponding time will increase exponentially, very fast. When the highest point is reached, this value will become the maximum throughput of the system. You can summarize the best throughput data of the product subsystem server by viewing the performance comparison and precautions before and after the number of connections, and viewing all the precautions and cross-comparisons of multiple latitude data at the same time.

Among them, the number of write connections in the executable file has doubled. This can better control the incremental impact of the number of connections, such as B1, 2, 4, 8, 16, 32, 64, 128, and 256. There are a total of 9 levels that grow in parallel. At this time, you will find that the maximum request of the script is always specified as 120 seconds for the limit.

After social investigation, it was found that the focus of the security test is most malicious attacks on illegal requests. Malicious persons can directly infiltrate the server system by forging malicious URLs and launching system attacks. When testing, use simulating malicious requests from illegal personnel and spoofing URLs to further check whether your requests are successfully sent to the server. At the same time, you can simulate false submissions to check whether the system filtering method can be implemented correctly on the system application. If you want to know the current security accurately, these methods can be applied very well. During the testing process, the items and results are very important. During the test, many typical malicious attacks were simulated, such as SQL, XSS, and header CLRF. These can bypass the INFO loophole and forge a fake demand. As a result, we can see that all malicious requests have failed and all subsystems can withstand security vulnerabilities and fully achieve the expected test goals.

Functional testing shows that the product subsystem can meet all expected development needs, meet all need analysis goals, develop in accordance with industry specifications, and develop in strict accordance with the company's software development history. It has an excellent user experience and a good interface.

Compatibility testing can use basic functions to normally display product subsystems and display transactions in today's conventional browsers. All browsers are displaying and operating normally, no phenomena such as incompatibility have been found, and the fundamental requirements for compatibility have been met.

Through the system performance test, the product subsystem has reached a throughput of 999TPS under the best hardware and network conditions, and 91% of the response time is less than 999 ms. This utilization rate has achieved the expected requirements. The ability to fully meet the requirements of high flow and high parallelism indicates that the product subsystem has achieved its expected performance goals.

## 5. Conclusion

The product subsystem of the b2c e-commerce platform uses a platform to integrate multiple product lines of the company's sales business. It provides business users with safe and convenient one-stop transaction support for the products they need, and businesses continue to add new products. This paper analyzes the current development of the b2c e-commerce platform and the needs of the product subsystem of the e-commerce platform, and successfully completed its system architecture design. Refer to the Spring MVC server architecture, summarize the design of six modules: login and registration, product release management, product transaction, product recommendation selection, search order display, and product purchase, and finally realize the application of detailed inspection and test schemes for product subsystems. Shopping lists, behavior analysis, and shopping suggestions for users are important parts of building a shopping website. To activate these modules, a large amount of historical data is required. The analysis concluded that the influence of their behavior and rules is far greater than the influence of various algorithms. If you want to make full use of such a large amount of data, then the first requirement for these analyses is to effectively access such a large amount of data. In the context of the Internet, data are precious. If you want to tap more wealth, you need more data. After analyzing a large amount of data and using different logics to process it, a lot of valuable things will be discovered. Information is a good guide for future decisions and instructions so that the company can better formulate its business strategy.

## Figures and Tables

**Table 1:** Comparison of search efficiency of two repair algorithms for different *p* value RDP.

*p*	Exhaustive traversal (ms)	CHR (ms)	Improvement ratio (%)
5	0.0220	0.0100	54.55
7	0.0950	0.0310	6737
11	2.3160	0.3910	83.12
13	11.9840	1.6150	86.52
17	107.7410	10.0790	90.65
19	455.2760	40.5370	91.10
23	9230.7800	691.2800	92.51
29	752296.2700	45423.5570	93.93

**Table 2:** The probability that the CHR algorithm provides the global minimum repair cost.

*p*	Probability of hitting the global optimum (%)	Global optimization is the biggest improvement over CHR (%)
5	94.9	6.12
7	94.5	5.54
11	93.6	5.98
13	93.2	6.46
17	92.8	5.97
19	93.1	5.73

**Table 3:** Comparison of time complexity of different algorithms.

Algorithms	Time complexity
Weighted-KM-Slope-Vu	*k*·*m*·*t*·*f* + *k*·*n*·*n*
SVD	*t*·*m*·*n*·*d*
SVD++	*t*·*in*·*n*·*d*

**Table 4:** DC product.

Field name	Types of	Length	Empty or not	Description
Id	INTEGER	No	No	Id
Piroduct_code	CHAR	15	No	Product code
Categoiy_id	CHAR	10	No	Product type
Brand_id	CHAR	10	Air	Brand id
Product_title	Varchar	500	Air	Product title
Subtitle	Varchar	500	Air	Subtitle
Bargaining_sales	CHAR	1	Air	Pricing sales
Product_relation_code	CHAR	10	Air	Product association code
Hide_bidden	CHAR	1	Air	Whether to hide the price (1 is hidden. 0 is not hidden)

**Table 5:** DC product category.

Field name	Types of	Length	Empty or not	Description
Id	INTEGER	No	No	Id
Category id	CHAR	10	No	Category id
Category name	Varchar	50	No	Category name
Parent id	CHAR	10	Air	Parent class id
Sort	INTEGER	No	Air	Sort
Valid	CHAR	1	Air	Is it effective
Relation	INTEGER	1	Air	Number of layers

**Table 6:** DC product buy evaluation.

Field name	Types of	Length	Empty or not	Description
Id	INTEGER	No	No	Id
Evaluation_id	CHAR	10	No	Evaluation id
buyers_id	CHAR	10	No	Buyer id
Product_code	CHAR	15	No	Product code
Evahiation_content	Varchar	1000	Air	Comment content
Evaluation_tinie	Varchar	19	Air	Evaluation time

**Table 7:** DC buy request bidden.

Field name	Types of	Length	Empty or not	Description
Id	INTEGER	No	No	Id
Bidden_code	CHAR	10	No	Quotation number
Buy_code	CHAR	10	No	Buying order number
Bidden_time	CHAR	19	Air	Time quotes
Bidden_total	NUMBER (15.2)	2	Air	Total offer
Status	CHAR	2	Air	Status
End_time	CHAR	19	Air	Valid period

## Data Availability

The data used to support the findings of this study are available from the corresponding author upon request.
